# Quantitative MRI changes in neck muscle size and composition following long‐duration spaceflight

**DOI:** 10.1113/EP093726

**Published:** 2026-09-24

**Authors:** Diana A. Madrid Fuentes, Marina S. Sawires, Leon Lenchik, Janet A. Tooze, Ashley A. Weaver

**Affiliations:** ^1^ Department of Biomedical Engineering, Center for Injury Biomechanics Wake Forest University School of Medicine Winston‐Salem North Carolina USA; ^2^ Department of Radiology Stanford University School of Medicine Stanford California USA; ^3^ Department of Biostatistics and Data Science Wake Forest University School of Medicine Winston‐Salem North Carolina USA

**Keywords:** aerobic exercise, cervical muscle, Dixon magnetic resonance imaging, muscle atrophy, resistance training, spaceflight

## Abstract

Long‐duration spaceflight poses several challenges to human health, including muscle loss and fat deposition. To mitigate these effects, exercise countermeasures are prescribed. This study aimed to measure neck muscle size and composition changes with long‐duration spaceflight using quantitative magnetic resonance imaging (MRI) and examine associations with in‐flight exercise modality and volume. Nine astronauts (mission duration: 7.1 ± 1.8 months) underwent pre‐ and post‐flight 3T Dixon MRI of the cervical spine. Cross‐sectional area (CSA) and muscle fat fraction (MFF) were measured bilaterally in 11 neck muscles. Mixed‐effects models assessed monthly percentage changes and associations with resistance (ARED), cycle ergometer and treadmill training volumes. The CSA and MFF of most neck muscles did not change across long‐duration missions. CSA increased for the sternocleidomastoid (+1.21%/month, *P* = 0.004) and the rhomboid minor (+1.71%/month, *P* = 0.057). Semispinalis capitis MFF increased (+1.10%/month, *P* = 0.051). More ARED repetitions of different exercises were associated with CSA increases in four muscles (*P* < 0.001–0.097) and MFF reductions in three muscles (*P* = 0.040–0.093). More cycle ergometer time was associated with a CSA increase in the sternocleidomastoid (*P* = 0.053) and an MFF increase in the scalenus medius (*P* = 0.019), while treadmill time was linked to CSA decreases in the trapezius (*P* = 0.095) and rhomboid minor (*P* = 0.047). Neck muscle size and composition were generally preserved during modern long‐duration missions in which structured exercise countermeasures were performed. Because all crewmembers completed in‐flight exercises, these findings should not be interpreted as evidence that exercise countermeasures prevented neck muscle changes.

## Abstract (highlights)

- **What is the central question of this study?**
  How does long‐duration spaceflight affect neck muscle size and composition, and what are the associations between these muscle changes and in‐flight exercise exposure?
- **What is the main finding and its importance?**
  There was no evidence of decline in neck muscle size and composition following long‐duration spaceflight. Most neck muscles showed no significant changes in cross‐sectional area or muscle fat fraction, although the semispinalis capitis exhibited increased muscle fat fraction. These findings provide quantitative MRI data characterizing neck muscle changes following modern long‐duration missions and support ongoing efforts to optimize countermeasures.

## 1 INTRODUCTION

The National Aeronautics and Space Administration (NASA) is currently conducting the Artemis programme, a series of missions to the Moon intended to advance the long‐term goal of sending humans to Mars as early as the 2030s (National Aeronautics & Space Administration, 2025). Since a mission to Mars would require roughly 3 years of spaceflight (Portree, 2001), it is important to design effective countermeasures to mitigate the effects of such prolonged exposure to microgravity in the human body, which include primarily renal stones, urinary retention, neuro‐ocular syndrome, orthostatic intolerance, reduced aerobic capacity, bone changes and muscle declines (Valinia et al., 2022). Minimizing the deleterious effects of microgravity on muscle is important since it not only endangers the health of crewmembers and their physical ability to complete their mission, but may also have irreversible long‐lasting effects upon re‐introduction to gravity (Tomsia et al., 2024). In line with this, the National Research Council called for translational research to elucidate the mechanisms for muscle structural deficits and to measure the effectiveness of countermeasures in correcting those deficits (National Research Council, 2011). One of the fronts through which muscle atrophy has been tackled is in‐flight exercise. Modern equipment includes the cycle ergometer with vibration isolation system (CEVIS), the second‐generation treadmill (T2) and most importantly the advanced resistance exercise device (ARED, which has provided capabilities for a wider range of exercises and enabled the use of heavier weights (Lamoreaux & Landeck, 2006). There is a need to understand whether modern exercise countermeasures are sufficient or if further modifications need to be made prior to multi‐year expeditions.

Of the cases of in‐mission back pain reported in astronauts, 11% are in the neck region; astronauts are also at increased risk of cervical intervertebral disc herniation after spaceflight (Chang et al., 2016; Johnston et al., 2010). The cause of this is not yet clear. On one hand, evidence from terrestrial analogues to spaceflight does not demonstrate significant neck muscle atrophy (Belavý et al., 2013); on the other hand, studies suggest that muscle size and quality may play a role in neck pain and risk of intervertebral disc herniation under other adverse circumstances (Elliott et al., 2006; Li et al., 2025). The deposition of adipose tissue in skeletal muscle has gained attention as a relevant mechanism for functional impairment and is associated with disability in a variety of conditions (Addison et al., 2014; Beavers et al., 2013; Grondin et al., 2022; Huynh et al., 2022; Snodgrass et al., 2024). Although our group has previously examined changes in neck muscle size with spaceflight, that retrospective study used the pixel intensity ratio of muscle to fat from T1 magnetic resonance imaging (MRI) (McNamara et al., 2019). T1 image contrast is generated through the differences in T1 relaxation time, which is not directly tied to muscle fat content; furthermore, referencing fat tissue, even from the same image section, can introduce inaccuracies due to field bias (Burakiewicz et al., 2017; Huber et al., 2020). A more accurate method is the chemical shift Dixon MRI, which generates dedicated fat and water images exploiting the higher resonance frequency of hydrogen nuclei in water molecules than in fat molecules. Dixon MRI has been well‐established as an accurate, reproducible and sensitive way of measuring fat deposition in muscle (Lenchik et al., 2024).

The aim of this study was primarily to investigate the effect of long‐duration spaceflight on change in neck muscle size and composition of crewmembers undergoing missions of 6+ month duration in the last decade. Secondarily we aimed to examine the association between changes in neck musculature and in‐flight exercise modality and volume.

## 2 METHODS

### 2.1 Ethical approval

This study was approved by the Institutional Review Boards of Wake Forest University School of Medicine (approval number: IRB00035882) and NASA (approval number: Pro2280). Written informed consent was obtained from each crewmember. The study conformed to the standards set by the *Declaration of Helsinki*.

### 2.2 Study sample and imaging protocol

This study used data obtained from NASA. The sample consisted of nine subjects who underwent long‐duration spaceflight missions (≥6 months), including an approximately balanced number of male and female subjects (ages 44 ± SD 4.6 years), with an average mission duration 7.1 ± SD 1.8 months.

All crewmembers underwent a pre‐flight and post‐flight scan of their entire spine using a two‐point Dixon volumetric interpolated breath‐hold examination protocol with a 3T MRI scanner (Siemens Verio, Erlangen, Germany). Dixon techniques deliver in‐phase and opposed‐phase images for water and fat molecules, from which water‐only and fat‐only images are obtained by the scanner. These water and fat images are correlated to the concentration of mobile protons of water and of triglycerides, respectively. The acquisition protocol was repetition time/time to echo of 6.73/1.35 ms, flip angle 10°, pixel spacing of 1.2125/1.2125 mm, slice thickness of 1.2 mm, 208 slices, 320 columns and 250 rows. Pre‐flight scans took place 41–166 days before flight and post‐flight scans took place 4–10 days after returning. The average time between scans is 10.9 ± SD 1.9 months.

### 2.3 Muscle segmentation and imaging data collection

**FIGURE 1:**
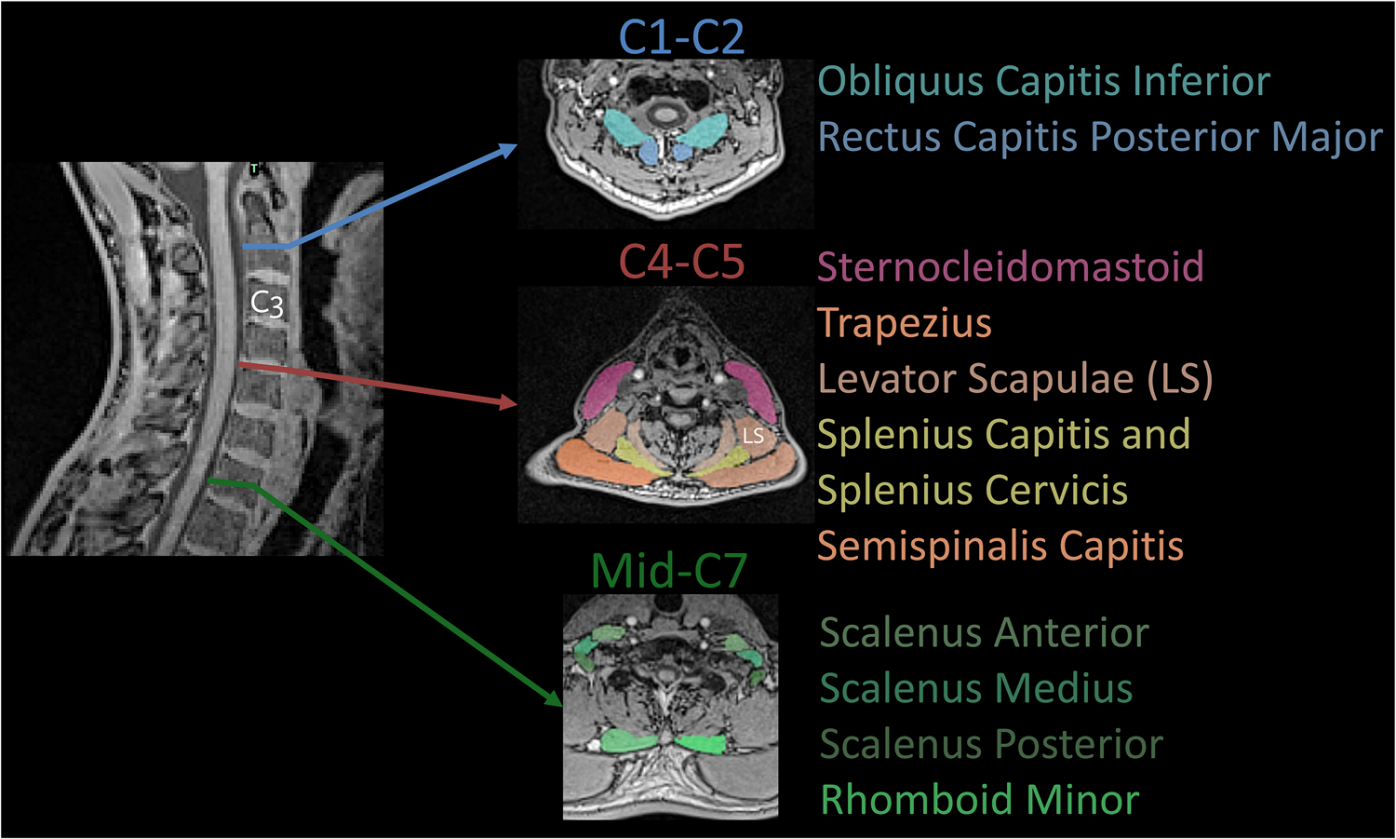
Neck muscles analysed.

An operator (M.S.) was trained to perform the initial muscle segmentation, after which the segmentations underwent final refinement and revision by D.M. who made the final estimates. Segmentations were manually performed using commercial image processing software (Materialise Mimics version 23, Leuven, Belgium). The analysts were not provided with information regarding the participant, mission durations or exercise exposure during the segmentation process. The segmentation procedure was based on predefined anatomical boundaries that were applied consistently across all scans (McNamara et al., 2019). Muscle segmentations are shown in Figure 1. From the C1–C2 intervertebral disc level, the obliquus capitis inferior and rectus capitis posterior major muscles were segmented. From the C4–C5 intervertebral disc level, the levator scapulae, semispinalis capitis, splenius capitis and cervicis (together), sternocleidomastoid and trapezius muscles were segmented. From the mid‐C7 level, the scalenus anterior, scalenus medius, scalenus posterior and rhomboid minor muscles were segmented. All segmentations were bilateral.

Muscle cross‐sectional area (mm^2^) and average muscle fat fraction (MFF, %) were determined for all neck muscles at the previously described cervical levels. MFF, also known as proton density fat fraction, was calculated by dividing the mean signal intensity of the fat image by the summed signal intensity of the fat and water images (MFF = fat/(fat + water) × 100), ranging from 0 to 100%, which represents the proportion of fat protons relative to the combined signal of fat and water protons. Higher MFF indicates a greater amount of fat within the muscle tissue.

### 2.4 Weight and in‐flight exercise data

Weight (kg) was extracted from the body mass measurement device (BMMD) logs using the first and last record available. In‐flight CEVIS and T2 data were available for nine subjects. ARED data were only available for six of the nine crew members. The following ARED exercises were selected for analysis due to their consistent logging across crewmembers, due to their compound nature and potential influence on neck muscles: bent over rows, deadlifts, Romanian deadlifts, shoulder shrug, squats (back squats, single leg squats), sumo squats and upright rows. Exercise logs included the exercises performed with the ARED with session date, number of reps and sets; time utilizing the cycle ergometer CEVIS; and time utilizing the T2 treadmill. The T2 includes a bungee system attached to a harness worn by the user to counter the microgravity environment. The CEVIS also has a harness with additional straps on the pedals; it works through a standard flywheel and braking‐band system to generate the resistance (NASA Life Sciences Portal). The ARED utilizes flywheels and piston‐driven vacuum cylinders to provide mechanical resistance and simulate the inertial loading of free‐weights (Lamoreaux & Landeck, 2006).  The total time spent on CEVIS and T2 and the number of repetitions per exercise on ARED were normalized by individual mission duration. All warmup repetitions were excluded from analysis.

### 2.5 Statistical analysis

Statistical analysis was performed using SAS (version 9.4, SAS Institute Inc., Cary, NC, USA) and JMP (JMP Pro v17, SAS Institute Inc.). Model‐based estimates are reported with standard errors and confidence intervals. Exact *P*‐values are reported to three significant figures, except when *P* < 0.0001.

Given the small number of available participants inherent to astronaut cohorts, the α‐level was prespecified at 0.10 to reduce the likelihood of overlooking potentially meaningful spaceflight‐related changes. This threshold is more liberal than the conventional α‐level of 0.05 and increases the likelihood of false‐positive findings. Therefore, estimates, standard errors, confidence intervals and exact *P*‐values are emphasized throughout. Ninety percent confidence intervals were calculated to correspond to the prespecified two‐sided α‐level of 0.10.

We computed the change of body mass, muscle area and MFF by calculating the difference of follow‐up minus baseline, divided by baseline, and then divided by length of mission duration. Change in body weight was assessed with a one‐sample Student's *t*‐test.

The primary CSA and MFF analyses included nine crewmembers. Because each muscle was measured bilaterally, mixed‐effects models were used to account for repeated measurements within crewmembers. Mixed models were used to estimate muscle area and MFF monthly changes with subject and side as random effects. For muscles with statistically detectable CSA or MFF changes, additional mixed models were used to evaluate whether monthly percentage change in the body mass was associated with monthly percentage change in the muscle outcome. In these models, body mass monthly percentage change was included as a fixed effect, with subject and side included as random effects.

Exercise association analyses were intended to generate hypotheses regarding potential associations between in‐flight exercise exposure and neck muscle adaptation during long‐duration spaceflight. ARED analyses included the six crewmembers with those available data. Mixed models were used to measure the association between monthly percentage changes in muscle size and MFF and every 100 repetitions of each ARED exercise. Subject and laterality were included as random effects, and ARED exercise was included as a fixed effect.

CEVIS and T2 analyses included the nine crewmembers with available aerobic exercise data. Separate mixed models were used to evaluate the association between monthly percentage change in CSA or MFF and every hour spent on CEVIS or T2, with CEVIS and T2 time normalized by individual mission duration. CEVIS or T2 exposure was included as a fixed effect, with subject and side included as random effects. The exercise association models evaluated prespecified muscle–exercise pairs based on the segmented neck muscles and available in‐flight exercise exposures. Because many prespecified associations were evaluated, the possibility of false‐positive findings is increased. Accordingly, these results are interpreted as hypothesis‐generating for future studies and spaceflight physiology models rather than definitive evidence for any single exercise‐specific effect.

To assess segmentation reliability, D.M. repeated the process in a blinded fashion on a replicate for four muscles: obliquus capitis inferior, rectus capitis posterior major, levator scapulae, and splenius capitis and cervicis. Intra‐rater reliability was assessed by calculating intraclass correlations (ICC) of the repeated measurements for four participants from mixed models that adjusted for the time point of the repeated measurement (pre, post) as a random and fixed effect. Four muscles were selected a priori to represent different anatomical levels, muscle sizes, and segmentation complexities within the neck. Repeat segmentations were conducted while blinded to the original segmentation and time point. An ICC of 0.75–0.90 was considered good intra‐rater reliability and ICC > 0.90 was considered excellent.

## 3 RESULTS

**FIGURE 2:**
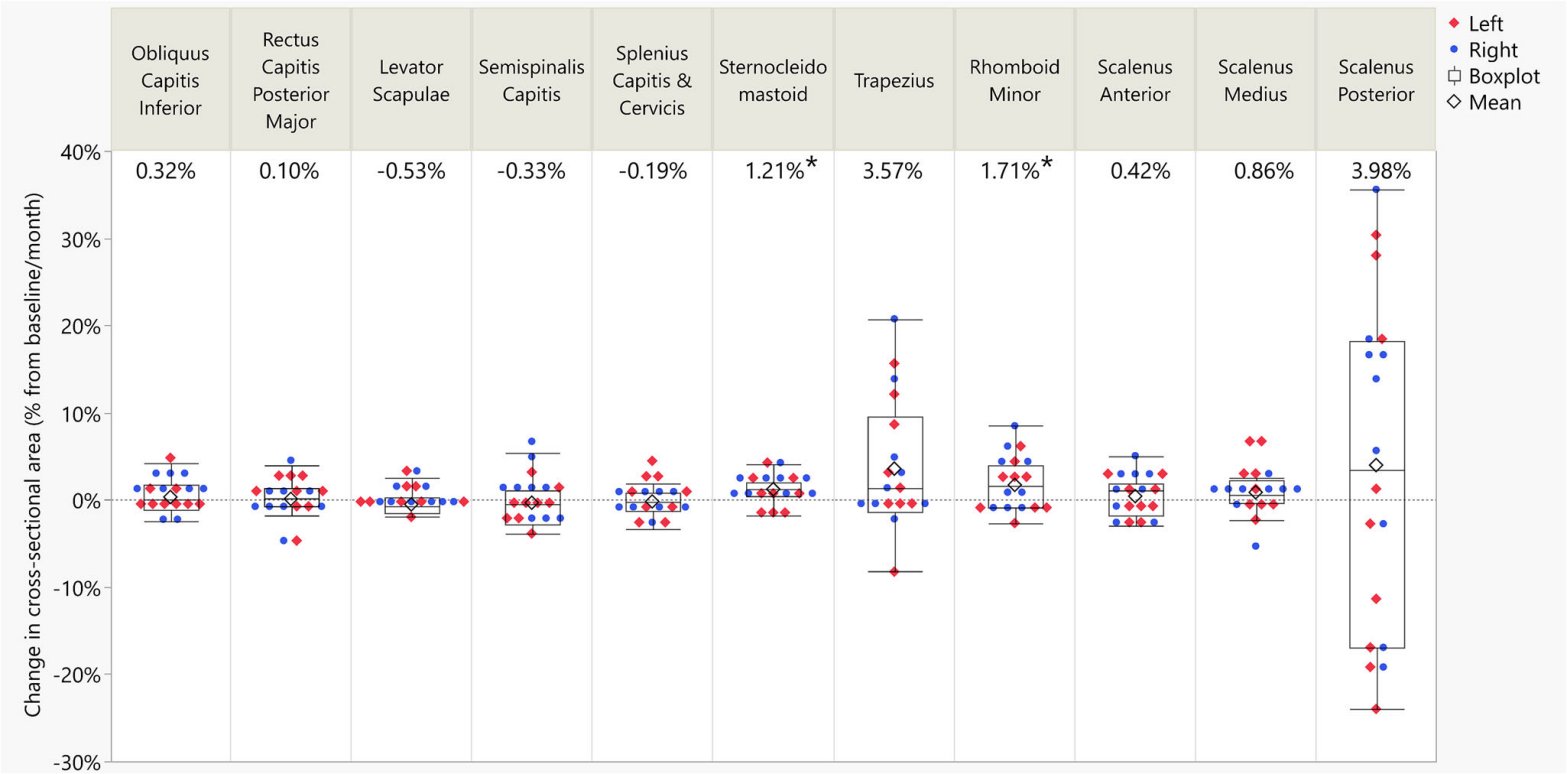
Muscle cross‐sectional area change expressed as percentage from baseline per month of mission duration. The whiskers mark the minimum and maximum data values excluding outliers, the centre line is the median and the top and bottom of the box are the 75th and 25th percentiles, respectively. The mean is represented with a rhombus. *n* = 9 participants contributed 18 bilateral muscle measurements.

**FIGURE 3:**
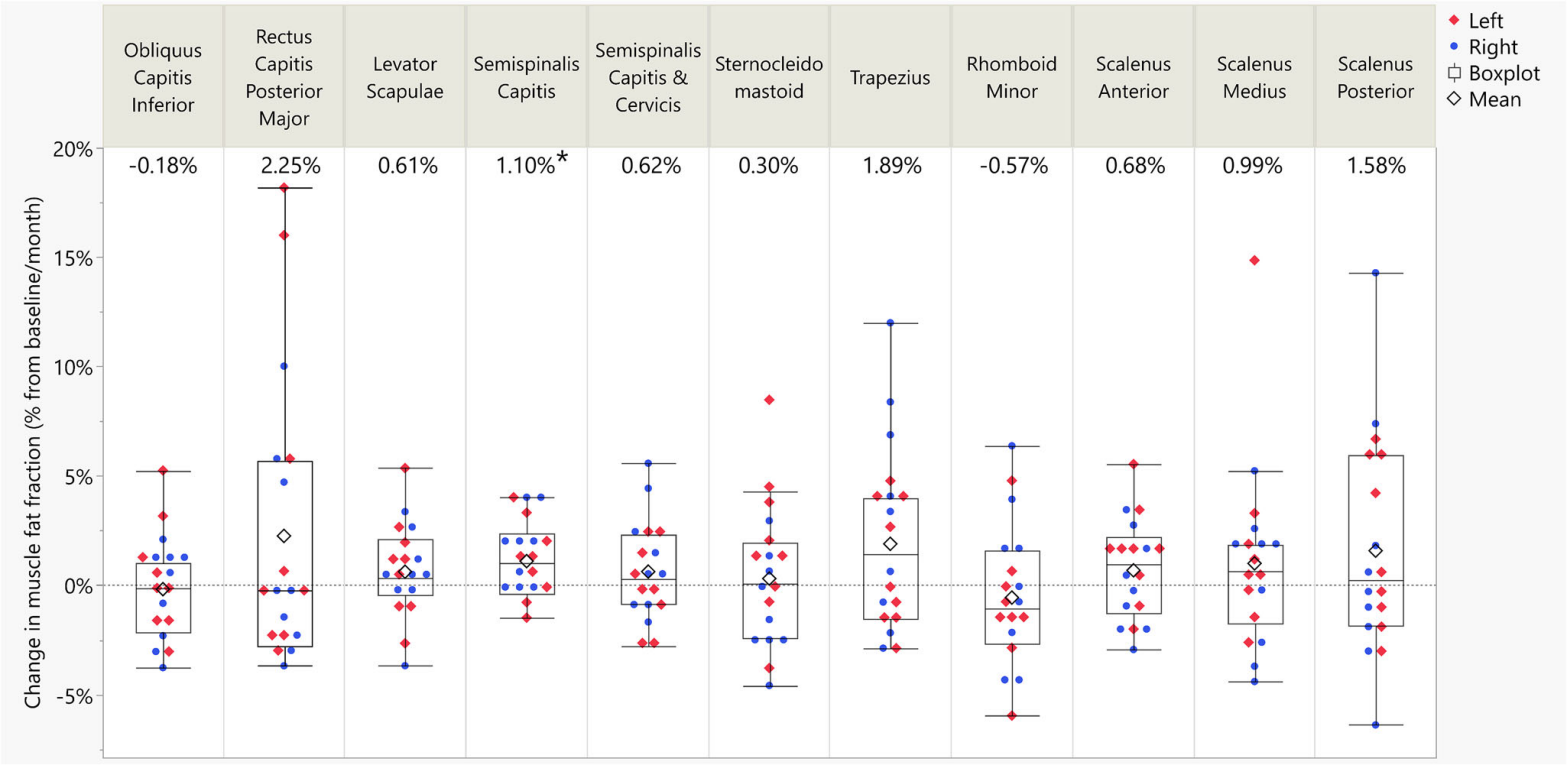
Muscle fat fraction change expressed as percentage from baseline per month of mission duration. The whiskers mark the minimum and maximum data values excluding outliers, the centre line is the median and the top and bottom of the box are the 75th and 25th percentiles, respectively. The mean is represented with a rhombus. The number of datapoints is 18, each representing a left and right side measurement for each crewmember.

Body mass from baseline changed 0.19%/month (*P* = 0.16, *n* = 9). Most muscles exhibited no change in muscle cross‐sectional area, except for the sternocleidomastoid whose area increased 1.21% per month of mission duration (SE = 0.41; 90% CI: 0.54 to 1.88; *P* = 0.004, *n* = 9 participants contributed 18 bilateral muscle measurements) and the rhomboid minor whose area increased 1.71% per month (SE = 0.89; 90% CI: 0.25 to 3.17; *P* = 0.057, *n* = 18) (Table 1, Figure 2). Most muscles exhibited no change in MFF, except for the semispinalis capitis that increased 1.10% (SE = 0.56; 90% CI: 0.18 to 2.02; *P* = 0.051, *n* = 9 participants contributed 18 bilateral muscle measurements) per month of mission duration (Table 1, Figure 3). Body mass monthly percentage change was not associated with sternocleidomastoid area percentage change (β = 0.55; SE = 1.46; 90% CI: −2.21 to 3.31; *P* = 0.717, *n* = 9 participants contributed 18 bilateral muscle measurements), with rhomboid minor area percentage change (β = 2.42; SE = 2.96; 90% CI: −3.18 to 8.02; *P* = 0.441, *n* = 9 participants contributed 18 bilateral muscle measurements) or with semispinalis capitis MFF percentage change (β = −2.38; SE = 1.44; 90% CI: −5.10 to 0.35; *P* = 0.142, *n* = 9 participants contributed 18 bilateral muscle measurements).

**TABLE 1:** Muscle cross‐sectional area change expressed as a percentage from baseline per month of mission duration.

	Cross‐sectional area				Muscle fat fraction			
Neck muscle	Monthly percentage change	standard error	90% confidence interval	*P* ^*^	Monthly percentage change	Standard error	90% confidence interval	*P*
Obliquus capitis inferior	0.32	0.47	−0.45 to 1.09	0.495	−0.18	0.67	−1.28 to 0.92	0.786
Rectus capitis posterior major	0.10	0.78	−1.19 to 1.39	0.900	2.25	1.69	−0.52 to 5.03	0.185
Levator scapulae	−0.53	0.37	−1.14 to 0.09	0.161	0.61	0.69	−0.53 to 1.75	0.380
Semispinalis capitis	−0.33	0.94	−1.88 to 1.21	0.723	1.10	0.56	0.18 to 2.01	0.051^*^
Splenius capitis and cervicis	−0.19	0.48	−0.98 to 0.61	0.703	0.62	0.60	−0.36 to 1.61	0.302
Sternocleidomastoid	1.21	0.41	0.53 to 1.89	0.004^*^	0.30	0.78	−0.98 to 1.58	0.700
Trapezius	3.57	2.27	−0.16 to 7.31	0.119	1.89	1.15	−0.00 to 3.78	0.104
Rhomboid minor	1.71	0.89	0.25 to 3.17	0.057^*^	−0.57	1.09	−2.36 to 1.22	0.603
Scalenus anterior	0.42	0.81	−0.92 to 1.76	0.610	0.68	0.58	−0.28 to 1.64	0.249
Scalenus medius	0.86	0.77	−0.41 to 2.13	0.265	0.99	1.24	−1.04 to 3.03	0.424
Scalenus posterior	3.98	4.49	−3.41 to 11.37	0.377	1.58	1.41	−0.74 to 3.89	0.265

*Note*: The number of datapoints is 18, each representing a left and right side measurement for each crewmember.

* Significant at two‐sided *P* <  0.1.

**FIGURE 4:**
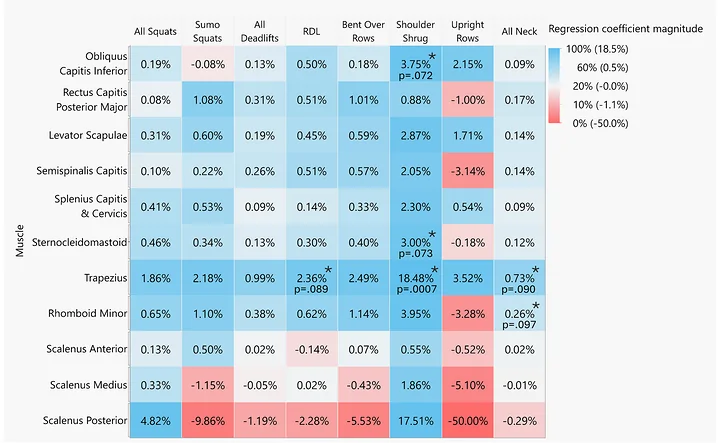
Changes in neck muscle cross‐sectional area vs. in‐flight ARED exercise. Each β‐coefficient indicates monthly percentage change per 100 reps of resistance exercise on ARED. *Statistically significant change at *P* < 0.1, based on the mixed model. RDL, Romanian deadlift. All Neck refers to the aggregate of all the previous exercises. *n* = 6 participants per association; each association has 12 datapoints including the left and right side muscle measurement for each crewmember.

For the six astronauts with ARED data, the average number of repetitions per session for all exercises was 243 ± 39, and 132 ± 24 for exercises included in the neck muscle analysis. The data for the linear mixed models is shown in Figure 4 and Appendix Table A1. Performing Romanian deadlifts was associated with area increases of the trapezius (β = 0.0236; 90% CI: 0.0045 to 0.0427; *P* = 0.089). Performing shoulder shrugs was associated with area increases of the obliquus capitis inferior (β = 0.0375; 90% CI: 0.0092 to 0.0658; *P* = 0.072), sternocleidomastoid (β = 0.0300; 90% CI: 0.0073 to 0.0527; *P* = 0.073) and trapezius (β = 0.1848; 90% CI: 0.1373 to 0.2323; *P* = 0.0007). Aggregated ARED exercise was associated with area increases of the trapezius (β = 0.0073; 90% CI: 0.0014 to 0.0132; *P* = 0.090) and the rhomboid minor (β = 0.0026; 90% CI: 0.0005 to 0.0047; *P* = 0.097).

**FIGURE 5:**
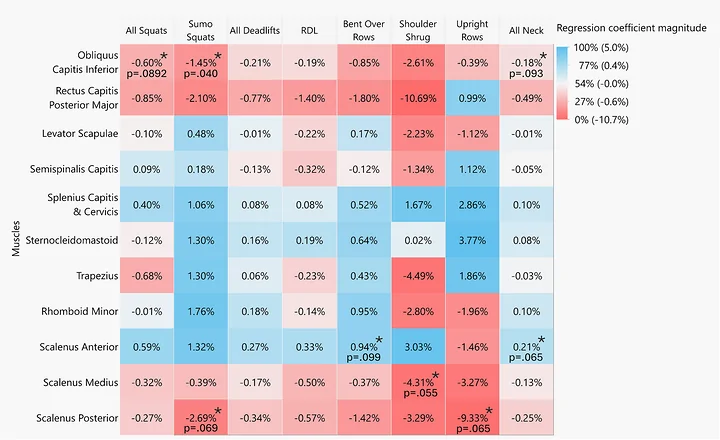
Changes in neck muscle fat fraction vs. in‐flight ARED exercise. Each β‐coefficient indicates monthly percentage change per 100 reps of resistance exercise on ARED. *Statistically significant change at *P* < 0.1, based on the mixed model. RDL, Romanian deadlift. All Neck refers to the aggregate of all the previous exercises. *n* = 6 participants per association; each association has 12 datapoints including the left and right side muscle measurement for each crewmember.

The linear mixed models between changes in MFF and every 100 repetitions indicate that resistance training in the ARED was associated with decreasing MFF in six instances and increasing MFF in two instances (Figure 5, Appendix Table A2). Specifically, decreases in MFF of the obliquus capitis inferior were associated with squats (β = −0.0060; 90% CI: −0.0107 to −0.0011; *P* = 0.089), sumo squats (β = −0.0145; 90% CI: −0.0237 to −0.0053; *P* = 0.040) and aggregated ARED exercise (β = −0.0018; 90% CI: −0.0033 to −0.0003; *P* = 0.093). Decreases in MFF of the scalenus medius were associated with shoulder shrugs (β = −0.0431; 90% CI: −0.073 to −0.0132; *P* = 0.055). Decreases in MFF of the scalenus posterior were associated with sumo squats (β = −0.0269; 90% CI: −0.0468 to −0.007; *P* = 0.069) and upright rows (β = −0.0933; 90% CI: −0.1586 to −0.0288; *P* = 0.065). Increases in MFF of the scalenus anterior were associated with bent‐over rows (β = 0.0094; 90% CI: 0.0015 to 0.0173; *P* = 0.099) and aggregated ARED exercise (β = 0.0021; 90% CI: 0.0006 to 0.0036; *P* = 0.065).

CEVIS usage was mean ± SD 83 ± 30 min per week, T2 usage was 89 ± 28 min per week and, jointly, total aerobic exercise time was 172 ± 38 min per week. The mixed model results for every muscle change for CEVIS and T2 are shown in Appendix Table A3.

For every CEVIS hour, sternocleidomastoid monthly muscle area percentage increased 0.41% (β = 0.0041; 90% CI: 0.0011 to 0.0071; *P* = 0.053). Additionally, more CEVIS time was associated with an increase in MFF of the scalenus medius (β = 0.013; 90% CI: 0.0056 to 0.020; *P* = 0.019).

For every T2 hour, the scalenus anterior monthly muscle area increased by 0.66% (β = 0.0066; 90% CI: 0.0018 to 0.014; *P* = 0.049). For every T2 hour, the following muscle areas decreased: rhomboid minor (β = −0.0097; 90% CI: −0.0167 to −0.0027; *P* = 0.047) and trapezius (β = −0.0212; 90% CI: −0.0399 to −0.0025; *P* = 0.095). No association was found between T2 usage and change in the MFF of the neck muscles.

Intra‐rater reliability was excellent for most muscles evaluated. ICCs were 0.98 and 0.99 for the left and right obliquus capitis inferior, 0.84 and 0.95 for the left and right rectus capitis posterior major, 0.96 and 0.99 for the left and right levator scapulae, and 0.93 and 0.99 for the left and right splenius capitis and cervicis. Seven of eight measurements demonstrated excellent reliability (ICC > 0.90), whereas the left rectus capitis posterior major demonstrated good reliability (ICC of 0.75 to 0.90).

## 4 DISCUSSION

Here we provide a prospective study of the muscle cross‐sectional area and MFF changes of the neck musculature and their association with exercise intensity and modality in long‐duration spaceflight. We observed that there were no significant declines in muscle area, with average monthly changes ranging from −0.5 to 4%. Regarding muscle fat fraction, only the semispinalis capitis experienced an increase (+1.1% from baseline per month), while all other muscles experienced no significant change (averages ranging from −0.2 to 2% per month). Change in body mass was not associated with the significant muscle area or MFF changes. We also found that shoulder shrugs using the ARED, the only exercise directed towards some neck muscles, was positively associated with changes in the muscle size of three neck muscles during spaceflight. These findings do not establish that in‐flight exercise countermeasures caused preservation of neck muscle size or composition, because all crewmembers completed structured exercise and no non‐exercising comparison group was available. However, the overall preservation of neck muscle CSA and MFF during these missions provides useful descriptive evidence of neck muscle before and after modern long‐duration spaceflight.

There are very few studies examining the effects of spaceflight in the neck muscles. Our group's previous retrospective report found no atrophy in the neck muscles of astronauts, and one suggestion was that using an imaging technique more suitable to evaluate muscle fat content, such as Dixon MRI, could help further clarify if modern exercise countermeasures maintained neck muscle composition (McNamara et al., 2019). The results presented here directly address that limitation and suggest that neck muscle health is mostly preserved in long‐duration spaceflight and describe that in several muscles more repetitions of resistance exercise are associated with positive changes in muscle, albeit with large variability.

Our accompanying analysis of the thoracolumbar muscles of this crewmember sample found that the cross‐sectional area declined in the paraspinal and the quadratus lumborum muscles, along with increasing MFF in the thoracic muscles (Poveda et al., 2025). In the present study, we did not find evidence of declines in muscle size, but MFF increased in the semispinalis capitis. A potential implication is the central and lower muscles of the body, but not the neck muscles, may experience more negative effects with spaceflight. In line with this, another ARED‐era report found that total lean mass and bench press strength was preserved in long‐duration missions, while leg press strength and quadriceps, hamstrings and calf cross‐sectional area were all below preflight levels (Scott et al., 2023). In our study, the one muscle that experienced increasing MFF was the semispinalis capitis, a long muscle that functions as a head and neck extensor and rotator. Higher muscle fat infiltration (+0.5% percentage points) of the semispinalis capitis has been associated in cross‐sectional studies with females with chronic whiplash‐associated disorder (Van Looveren et al., 2021), and its reduced size has been associated with chronic non‐specific neck pain in females (Rezasoltani et al., 2012); however, these studies are far from conclusive. The levels of muscle fat content that compromise health and disability are not well defined in terrestrial settings. In a cross‐sectional study of healthy men aged 20–70 years, thigh MFF increased by approximately 1.3% per year of age relative to the estimated value at age 20 (Engelke et al., 2022). Similarly, a large cross‐sectional study of adults aged 45–84 years found progressively greater intramuscular fat in the thigh with increasing age; based on the reported age‐group means, this corresponds to an approximately 0.9–1.2% relative increase per year of age (Anderson et al., 2025). Although both of these studies are cross‐sectional and examine different muscles, direct comparison would suggest that in the current study the semispinalis capitis was accruing a year of MFF increase per month of spaceflight.

Average total aerobic exercise time for the nine astronauts was 172 ± 38 min per week, with average time spent evenly between treadmill and cycle ergometer, although this ratio varied by individual. We do not know the duration of the resistance exercise sessions for each astronaut, but for the six astronauts with resistance training data, if each repetition (no warmup) is adjudicated a 5‐s duration and a ratio of rest time to rep time of 2:1 is observed (total 15 s per repetition), we can approximate that 367 ± 69 min per week was spent using the ARED. This total exercise time of ∼539 min per week is well within the 450–720 min per week previously reported in a larger astronaut sample (Scott et al., 2023). Astronauts will be exposed long‐term to space's microgravity and to the sub‐terrestrial gravitational acceleration of the Moon and Mars during future missions. These explorations will require physically demanding and time‐consuming tasks to be performed, such as landing, terrain exploration, building an environment, carrying material, climbing and other largely unaided extravehicular activities (Sutterfield et al., 2019). With those time‐ and energy‐intensive tasks on the horizon, and with the multisystem effects of reduced gravity in mind, additional countermeasures beyond traditional exercise should be studied and optimized, such as tailored nutritional plans, plyometric exercise, lower body negative pressure, anti‐inflammatory drugs and artificial gravity (Ashari & Hargens, 2020; Basner et al., 2021; Belavý et al., 2011; Chiaberge et al., 2025; Smith et al., 2020). A report shows that a high intensity, lower volume programme exhibits protective effects comparable to the traditional low intensity, high volume programmes (English et al., 2020), and future research could apply the imaging methods herein to examine the effect of this new regime in muscle health.

There is little information on the effect of different exercise modalities on the neck muscles on Earth. Nevertheless, resistance exercise has been found to be the most effective modality to elicit hypertrophy (Grgic et al., 2019; Schoenfeld et al., 2021). Additionally, in certain settings, such as during weight loss, resistance exercise is more effective than aerobic training in minimizing muscle fat deposition (Madrid et al., 2023). In the present study, performing shoulder shrugs, the only isolation exercise in our analysis, was significantly associated with large increases in muscle area of the trapezius. Estimated associations between ARED exposure and CSA were positive for most evaluated muscle–exercise pairs, but the estimates were imprecise and these patterns require confirmation. Importantly, most exercises were not designed to target the neck, except shoulder shrugs.

For aerobic exercise, CEVIS time was associated with an increase in sternocleidomastoid muscle area, but also an increase in MFF in the scalenus medius. For T2, more exposure was associated with an increase in scalenus anterior muscle area and a decrease of rhomboid minor and trapezius muscle area. These modest findings are largely in line with the recent analysis of the thoracolumbar muscle, with T2 time being negatively associated with the longissimus thoracis area and negatively associated with quadratus lumborum MFF (Poveda et al., 2025). The lack of a consistent directionality across muscles and exercises may result from loading differences during treadmill running with harness systems, from different postural engagement or stabilization roles during cycling, from differential muscle fibre activation or muscle atrophy resistance. Regardless, the main purpose of the T2 treadmill and the CEVIS cycle ergometer is to train cardiorespiratory fitness, and the changes observed in neck muscle area and MFF do not imply that aerobic training greatly undermines muscle health. Further, a study on Earth in older adults undergoing weight loss, another scenario of complex mechanical unloading, suggests that combining aerobic and resistance training is best at reducing ectopic fat deposition (Waters et al., 2021).

There are many limitations in this study that should be considered when interpreting it. First, the small sample size that naturally occurs with human spaceflight research. Given the rare nature of the astronaut cohort and the small number of available participants, the α‐level was prespecified at 0.10 to reduce the likelihood of overlooking potentially meaningful spaceflight‐related changes that could inform future studies. While the approach is consistent with the hypothesis‐generating role of small‐sample human spaceflight studies, where estimates and confidence intervals may be more informative than reliance on a single dichotomous threshold, this threshold is more liberal than the conventional α‐level of 0.05 and increases the likelihood of false‐positive findings. In‐flight ARED data were only available for six out of nine subjects. All crewmembers performed structured in‐flight exercise; therefore, this study cannot determine how neck muscles would adapt to spaceflight in the absence of exercise in this modern setting, nor can it establish that preserved neck muscle size and composition were caused by the exercise prescription. We acknowledge that pre‐ and post‐flight images were not randomized prior to analysis. Because of the different mission durations and circumstances, while most scans were processed indistinctly of their timepoint, a few scans were processed as they were received, which limited the analyst's ability to be blinded to the temporal sequence of the data. However, the segmentation procedure was based on predefined anatomical boundaries that were applied consistently across all scans. In addition, all images were analysed using the same workflow and quality‐control procedures to ensure methodological consistency.

We do not delve into the potential effects of prehabilitation level in moderating spaceflight adaptation. Additionally, we did not consider the loads of each exercise into our analysis; however, astronauts are paired with a specialist years ahead of their mission and comprehensive training protocols are typically prescribed, in which selecting appropriate loads for hypertrophy and aerobic conditioning is addressed and taught to them (English et al., 2020; Scott et al., 2023). Finally, other groups have used convolutional neural networks to automatically segment individual neck muscle volume, while we used mainly a manual cross‐sectional area approach (Weber et al., 2021). Strengths of our work include an approximately equal sex distribution and the use of Dixon to quantify muscle fat fraction changes during ARED‐era missions.

Future research should examine whole muscle morphology changes as well as other mechanisms that might contribute to neck pain with microgravity, such as sensory changes (Sauer et al., 2023), intervertebral disc morphology and connective tissue properties. Research in such a direction is already on its way (Belavy et al., 2022). Future investigations should also incorporate randomized image ordering and fully blinded analyses whenever feasible. To further understand the unique biomechanical conditions associated with reduced gravity, other computational methodologies could be applied, such as simulations with musculoskeletal models, which have been successfully applied to the cervical region (He et al., 2025).

### 4.1 Conclusions

This study reports no consistent decline in neck muscle size or composition following modern long‐duration spaceflight. Because all crewmembers completed structured in‐flight exercise, these findings should not be interpreted as definitive evidence that exercise countermeasures prevented neck muscle changes. The observed associations between exercise exposure and muscle outcomes may help generate hypotheses for controlled studies and provide data for spaceflight physiology models examining regional muscle adaptation and countermeasure response for future long‐duration missions.

## AUTHOR CONTRIBUTIONS

Diana A. Madrid Fuentes and Marina S. Sawires contributed to data collection, data analysis and drafting of the manuscript. Janet Tooze contributed to the data analysis and drafting of the manuscript. Ashley A. Weaver, Leon Lenchik and Janet Tooze contributed to the study conception, study design, study oversight and editing of the manuscript. All authors have read and approved the final version of this manuscript and agree to be accountable for all aspects of the work in ensuring that questions related to the accuracy or integrity of any part of the work are appropriately investigated and resolved. All persons designated as authors qualify for authorship, and all those who qualify for authorship are listed.

## CONFLICT OF INTEREST

None declared.

## GENERATIVE AI STATEMENT

No generative artificial intelligence tool was used in the preparation of our manuscript.

## Data Availability

The datasets for this study will not be made publicly available because data belongs to the Life Sciences Data Archive and Lifetime Surveillance of Astronaut Health project.

## 1

**TABLE A1:** Regression coefficients β as percentage, standard errors (SE), and *P‐*values between monthly percentage changes in neck muscle size and in‐flight resistance exercise in the ARED

	All squats			Sumo squats			All deadlifts			Romanian deadlifts			Bent over rows			Shoulder shrug			Upright rows			All		
	β (SE)	90% confidence interval	*P*	β (SE)	90% confidence interval	*P*	β (SE)	90% confidence interval	*P*	β (SE)	90% confidence interval	*P*	β (SE)	90% confidence interval	*P*	β (SE)	90% confidence interval	*P*	β (SE)	90% confidence interval	*P*	β (SE)	90% confidence interval	*P*
Obliquus capitis inferior	0.2% (0.4)	−0.52 to 0.9	0.679	−0.1% (1)	−1.66 to 1.5	0.933	0.1% (0.2)	−0.2 to 0.46	0.528	0.5% (0.4)	−0.08 to 1.08	0.204	0.2% (0.7)	−0.91 to 1.27	0.791	3.8% (1.7)	0.92 to 6.58	0.072*	2.2% (3.1)	−2.88 to 7.18	0.513	0.1% (0.1)	−0.12 to 0.3	0.488
Rectus capitis posterior major	0.1% (0.6)	−0.82 to 0.98	0.893	1.1% (1.1)	−0.71 to 2.87	0.358	0.3% (0.22)	−0.05 to 0.67	0.200	0.5% (0.5)	−0.28 to 1.3	0.338	1% (0.7)	−0.09 to 2.11	0.180	0.9% (3.2)	−4.32 to 6.08	0.791	−1% (4.1)	−7.71 to 5.71	0.816	0.2% (0.2)	−0.08 to 0.42	0.275
Levator scapulae	0.3% (0.4)	−0.32 to 0.94	0.440	0.6% (0.8)	−0.78 to 1.98	0.503	0.2% (0.17)	−0.09 to 0.47	0.316	0.5% (0.3)	−0.09 to 0.99	0.219	0.6% (0.5)	−0.3 to 1.48	0.318	2.9% (1.8)	−0.16 to 5.9	0.171	1.7% (2.9)	−3.03 to 6.45	0.580	0.1% (0.1)	−0.02 to 0.3	0.225
Semispinalis capitis	0.1% (0.5)	−0.71 to 0.91	0.847	0.2% (1.1)	−1.54 to 1.98	0.843	0.3% (0.2)	−0.07 to 0.59	0.229	0.5% (0.4)	−0.16 to 1.18	0.259	0.6% (0.7)	−0.55 to 1.69	0.439	2% (2.6)	−2.27 to 6.35	0.465	−3.1% (3.3)	−8.52 to 2.24	0.380	0.1% (0.1)	−0.07 to 0.35	0.338
Splenius capitis and cervicis	0.4% (0.3)	−0.05 to 0.87	0.195	0.5% (0.7)	−0.56 to 1.62	0.453	0.1% (0.15)	−0.16 to 0.34	0.568	0.1% (0.3)	−0.35 to 0.63	0.652	0.3% (0.5)	−0.43 to 1.09	0.493	2.3% (1.6)	−0.38 to 4.98	0.207	0.5% (2.3)	−3.23 to 4.31	0.823	0.1% (0.1)	−0.06 to 0.24	0.332
Sternocleidomastoid	0.5% (0.3)	0.05 to 0.87	0.118	0.3% (0.7)	−0.84 to 1.52	0.651	0.1% (0.15)	−0.12 to 0.38	0.407	0.3% (0.3)	−0.19 to 0.79	0.353	0.4% (0.5)	−0.37 to 1.17	0.435	3.0% (1.4)	0.73 to 5.27	0.073*	−0.2% (2.5)	−4.31 to 3.95	0.945	0.1% (0.1)	−0.01 to 0.25	0.203
Trapezius	1.9% (1.4)	−0.44 to 4.16	0.234	2.2% (3.5)	−3.61 to 7.97	0.559	1% (0.64)	−0.06 to 2.04	0.174	2.4% (1.2)	0.45 to 4.27	0.089*	2.5% (2.2)	−1.16 to 6.14	0.305	18.5% (2.9)	13.73 to 23.23	<0.001*	3.5% (12.3)	−16.78 to 23.82	0.787	0.7% (0.4)	0.14 to 1.32	0.09*
Rhomboid minor	0.7% (0.5)	−0.22 to 1.52	0.266	1.1% (1.2)	−0.92 to 3.12	0.406	0.4% (0.23)	0 to 0.76	0.149	0.6% (0.5)	−0.24 to 1.48	0.281	1.1% (0.7)	−0.08 to 2.36	0.174	4% (3)	−0.9 to 8.8	0.230	−3.3% (4.3)	−10.3 to 3.74	0.477	0.3% (0.1)	0.05 to 0.47	0.097*
Scalenus anterior	0.1% (0.7)	−1.01 to 1.27	0.852	0.5% (1.5)	−1.95 to 2.95	0.749	0% (0.33)	−0.52 to 0.56	0.949	−0.1% (0.7)	−1.24 to 0.96	0.842	0.1% (1)	−1.64 to 1.78	0.949	0.6% (4)	−5.95 to 7.05	0.894	−0.5% (5.1)	−8.89 to 7.85	0.923	0% (0.2)	−0.33 to 0.37	0.909
Scalenus medius	0.3% (0.5)	−0.44 to 1.1	0.514	−1.2% (0.9)	−2.68 to 0.38	0.264	−0.1% (0.24)	−0.44 to 0.34	0.856	0% (0.5)	−0.79 to 0.83	0.975	−0.4% (0.7)	−1.63 to 0.77	0.577	1.9% (2.7)	−2.61 to 6.33	0.520	−5.1% (2.7)	−9.53 to −0.67	0.116	0% (0.2)	−0.26 to 0.24	0.957
Scalenus posterior	4.8% (4.1)	−1.86 to 11.5	0.280	−9.9% (9.1)	−24.81 to 5.09	0.320	−1.2% (2.2)	−4.81 to 2.43	0.607	−2.3% (4.5)	−9.7 to 5.14	0.632	−5.5% (6.6)	−16.37 to 5.31	0.433	17.5% (25.7)	−24.8 to 59.82	0.521	−50% (27)	−94.37 to −5.63	0.123	−0.3% (1.4)	−2.64 to 2.06	0.847

* *P *< 0.1.

**TABLE A2:** Regression coefficients β as percentage, standard errors (SE), and *P*‐values between monthly percentage changes in neck muscle fat fraction and in‐flight resistance exercise in the ARED

	All squats			Sumo squats			All deadlifts			Romanian deadlifts			Bent over rows			Shoulder shrug			Upright rows			All		
	β (SE)	90% confidence interval	*P*	β (SE)	90% confidence interval	*P*	β (SE)	90% confidence interval	*P*	β (SE)	90% Confidence interval	*P*	β (SE)	90% confidence interval	*P*	β (SE)	90% confidence interval	*P*	β (SE)	90% confidence interval	*P*	β (SE)	90% confidence interval	*P*
Obliquus capitis inferior	−0.6% (0.3)	−1.07 to ‐0.11	0.089*	−1.5% (0.6)	−2.37 to −0.53	0.040*	−0.2% (0.2)	−0.49 to 0.07	0.281	−0.2% (0.4)	−0.85 to 0.47	0.657	−0.9% (0.5)	−1.62 to −0.08	0.122	−2.6% (2)	−5.93 to 0.71	0.243	−0.4% (3.1)	−5.47 to 4.69	0.904	−0.2% (0.1)	−0.33 to −0.03	0.093*
Rectus capitis posterior major	−0.9% (1.5)	−3.25 to 1.55	0.583	−2.1% (3.2)	−7.3 to 3.1	0.531	−0.8% (0.6)	−1.79 to 0.25	0.263	−1.4% (1.3)	−3.57 to 0.77	0.329	−1.8% (2.1)	−5.29 to 1.69	0.429	−10.7% (6.9)	−22.09 to 0.71	0.174	1% (11.3)	−17.52 to 19.5	0.933	−0.5% (0.4)	−1.13 to 0.15	0.262
Levator scapulae	−0.1% (0.3)	−0.66 to 0.46	0.771	0.5% (0.7)	−0.7 to 1.66	0.531	0% (0.2)	−0.29 to 0.27	0.969	−0.2% (0.3)	−0.75 to 0.31	0.520	0.2% (0.5)	−0.69 to 1.03	0.756	−2.2% (1.6)	−4.93 to 0.47	0.224	−1.1% (2.5)	−5.22 to 2.98	0.672	0% (0.1)	−0.19 to 0.17	0.900
Semispinalis capitis	0.1% (0.3)	−0.45 to 0.63	0.783	0.2% (0.7)	−1 to 1.36	0.812	−0.1% (0.2)	−0.38 to 0.12	0.423	−0.3% (0.3)	−0.78 to 0.14	0.301	−0.1% (0.5)	−0.94 to 0.7	0.817	−1.3% (1.8)	−4.25 to 1.57	0.477	1.1% (2.4)	−2.8 to 5.04	0.658	−0.1% (0.1)	−0.21 to 0.11	0.639
Splenius capitis and cervicis	0.4% (0.3)	−0.14 to 0.94	0.271	1.1% (0.7)	−0.01 to 2.13	0.151	0.1% (0.2)	−0.22 to 0.38	0.656	0.1% (0.4)	−0.53 to 0.69	0.834	0.5% (0.5)	−0.34 to 1.38	0.359	1.7% (2.04)	−1.69 to 5.03	0.443	2.9% (2.5)	−1.17 to 6.89	0.295	0.1% (0.1)	−0.08 to 0.28	0.374
Sternocleidomastoid	−0.1% (0.4)	−0.84 to 0.6	0.787	1.3% (0.8)	0 to 2.6	0.15	0.2% (0.2)	−0.17 to 0.49	0.463	0.2% (0.4)	−0.52 to 0.9	0.680	0.6% (0.6)	−0.33 to 1.61	0.318	0.02% (2.6)	−4.19 to 4.23	0.995	3.8% (2.8)	−0.77 to 8.31	0.230	0.1% (0.1)	−0.13 to 0.29	0.541
Trapezius	−0.7% (0.9)	−2.09 to 0.73	0.461	1.3% (1.9)	−1.84 to 4.44	0.522	0.1% (0.4)	−0.66 to 0.78	0.895	−0.2% (0.9)	−1.71 to 1.25	0.809	0.4% (1.4)	−1.84 to 2.7	0.768	−4.5% (4.8)	−12.42 to 3.44	0.388	1.9% (6.8)	−9.28 to 13	0.795	−0.03% (0.3)	−0.49 to 0.43	0.917
Rhomboid minor	−0.01% (0.8)	−1.26 to 1.24	0.988	1.8% (1.4)	−0.56 to 4.08	0.259	0.2% (0.4)	−0.41 to 0.77	0.637	−0.1% (0.8)	−1.37 to 1.09	0.862	1% (1.1)	−0.78 to 2.68	0.402	−2.8% (4.2)	−9.63 to 4.03	0.525	−2% (5.6)	−11.09 to 7.17	0.738	0.1% (0.2)	−0.28 to 0.48	0.684
Scalenus anterior	0.6% (0.3)	0.06 to 1.12	0.12	1.3% (0.7)	0.17 to 2.47	0.11	0.3% (0.2)	0.01 to 0.53	0.13	0.3% (0.4)	−0.3 to 0.96	0.415	0.9% (0.5)	0.15 to 1.73	0.099*	3% (1.9)	−0.13 to 6.19	0.167	−1.5% (3)	−6.4 to 3.48	0.646	0.2% (0.1)	0.06 to 0.36	0.065*
Scalenus medius	−0.3% (0.4)	−1.04 to 0.4	0.500	−0.4% (1)	−2.05 to 1.27	0.709	−0.2% (0.2)	−0.52 to 0.18	0.464	−0.5% (0.4)	−1.14 to 0.14	0.249	−0.4% (0.7)	−1.51 to 0.77	0.611	−4.3% (1.8)	−7.3 to ‐1.32	0.055*	−3.3% (3.1)	−8.32 to 1.78	0.335	−0.1% (0.1)	−0.34 to 0.08	0.356
Scalenus posterior	−0.3% (0.8)	−1.62 to 1.08	0.754	−2.7% (1.2)	−4.68 to −0.7	0.069*	−0.3% (0.4)	−0.93 to 0.25	0.375	−0.6% (0.8)	−1.82 to 0.68	0.481	−1.4% (1.03)	−3.11 to 0.27	0.215	−3.3% (4.5)	−10.63 to 4.05	0.489	−9.3% (3.97)	−15.86 to −2.8	0.065*	−0.3% (0.2)	−0.61 to 0.11	0.307

* Indicates *P *< 0.1.

**TABLE A3:** Regression coefficients β as percentage, standard errors (SE), and *P*‐values between monthly percentage changes in neck muscle area and fat fraction and in‐flight cycle ergometer (CEVIS) and treadmill (T2).

	Cross‐sectional area						MFF					
	CEVIS			T2			CEVIS			T2		
	β (SE)	90% confidence interval	*P*	β (SE)	90% confidence interval	*P*	β (SE)	90% confidence interval	*P*	β (SE)	90% confidence interval	*P*
Obliquus capitis inferior	0.13 (0.23)	−0.25 to 0.51	0.574	−0.02 (0.26)	−0.44 to 0.4	0.945	0.18 (0.34)	−0.37 to 0.73	0.604	−0.56 (0.31)	−1.07 to −0.05	0.107
Rectus capitis posterior major	0.53 (0.33)	−0.01 to 1.07	0.139	−0.53 (0.38)	−1.15 to 0.09	0.195	0.89 (0.86)	−0.52 to 2.3	0.329	−1.38 (0.87)	−2.81 to 0.05	0.145
Levator scapulae	0.14 (0.2)	−0.18 to 0.46	0.506	−0.33 (0.18)	−0.63 to −0.03	0.102	−0.32 (0.29)	−0.79 to 0.15	0.291	0.03 (0.34)	−0.53 to 0.59	0.930
Semispinalis capitis	−0.09 (0.45)	−0.82 to 0.64	0.841	−0.32 (0.48)	−1.1 to 0.46	0.519	−0.02 (0.28)	−0.48 to 0.44	0.951	−0.13 (0.3)	−0.63 to 0.37	0.669
Splenius capitis and cervicis	−0.26 (0.2)	−0.59 to 0.07	0.228	−0.12 (0.23)	−0.51 to 0.27	0.623	0.41 (0.33)	−0.13 to 0.95	0.247	−0.21 (0.39)	−0.85 to 0.43	0.606
Sternocleidomastoid	0.41 (0.19)	0.11 to 0.71	0.053*	−0.35 (0.23)	−0.73 to 0.03	0.167	0.01 (0.38)	−0.62 to 0.64	0.983	−0.01 (0.42)	−0.7 to 0.68	0.975
Trapezius	−0.22 (1.26)	−2.29 to 1.85	0.864	−2.12 (1.13)	−3.99 to −0.25	0.095*	0.64 (0.57)	−0.3 to 1.58	0.289	0.68 (0.63)	−0.36 to 1.72	0.312
Rhomboid minor	0.61 (0.46)	−0.14 to 1.36	0.215	−0.97 (0.42)	−1.67 to −0.27	0.047*	0.14 (0.5)	−0.69 to 0.97	0.787	0.29 (0.54)	−0.6 to 1.18	0.603
Scalenus anterior	−0.19 (0.34)	−0.75 to 0.37	0.586	0.66 (0.29)	0.18 to 1.14	0.049*	0.38 (0.26)	−0.05 to 0.81	0.176	−0.01 (0.33)	−0.55 to 0.53	0.977
Scalenus medius	0.56 (0.35)	−0.01 to 1.13	0.141	−0.02 (0.45)	−0.75 to 0.71	0.970	1.3 (0.45)	0.56 to 2.04	0.019*	0.02 (0.73)	−1.19 to 1.23	0.977
Scalenus posterior	−1.21 (2.26)	−4.92 to 2.5	0.605	1.34 (2.48)	−2.74 to 5.42	0.601	0.81 (0.68)	−0.31 to 1.93	0.267	−0.91 (0.74)	−2.13 to 0.31	0.252

* *P *< 0.1.
